# Annotations

**Published:** 1896-12-19

**Authors:** 


### ANNOTATIONS.

### Ices in Midwinter.

It is ladies who provide ices in midwinter for their guests; and, so far as a pretty wide and careful personal observation can be relied upon, it is ladies who eat the ices when provided. A man, be he young, middle-aged, or old, is rarely, if ever, seen partaking of an ice in the month of December. Is it, then, the fact that blood of woman is of a higher temperature than that of man; that her heart pulsates with greater vigour, that she is less subject to gastric cramps and distressing colics, or to the possibilities of mere collapse due to sudden lowering of the temperature of the body? Not one of these questions can be answered in the affirmative. The blood of woman, instead of being better able to resist the cold than that of man, is less able, the action of her heart is not so vigorous and sustained, her nervous system is much more sensitive, and her stomach more likely to be the subject of cramps, and the intestine of painful colics. Why is it, then, that wherever a party of human beings are assembled under the control and direction of the more conservative sex, whether the occasion be an at home, or a small dining party, or a public ball, ices anywhere tempt the female palate and are partaken of at the risk of the sufferings we have mentioned, and of others there is not space to mention? It can only be that the female mind is so poor in resources that ices having once been began, and having been found agreeable to ladies, are brought in as a matter of course, all the year round, although for the winter months they are as unsuitable for the stomach as a muslin dress would be for outside wear.

### Livingstone and the Mission of Medicine.

It is often forgotten that David Livingstone, the greatest of missionaries, and the most illustrious of all the pioneers of African civilisation, was a medical man—a doctor of medicine. At home, medicine is too often looked upon as a mere bread-and-butter art whereby, in return for small services in physics, a man may live in a genteel way on the smallest possible of wages. In Africa medicine is the civiliser of civilisers—often clearing the way even for the religious teacher; and showing itself to be immensely more capable of converting the fighting savage into the civilised workman than either war or trade. Medicine, we believe, has not herself, at any rate as yet, reared any monument to the memory of Dr. Livingstone; but the Church Missionary Society has; and the monument is exactly what would have commended itself before all things to Livingstone's judgment and high humanitarian purpose in life. It is a college; and a college which gives young men a training, not for the teaching of religion only, but for the practising of rudimentary medicine in the mission field. The Council of the College have courteously sent us their annual report. In it we are glad to read that the authorities of some of the principal general hospitals give the "Livingstone students" very large facilities for learning clinical work. The house surgeons at some of the hospitals take a very special interest in the work of the young men, and their help is found to be invaluable. There is at the college itself a considerable staff of competent and specially qualified lecturers. Dr. Clapton is particularly mentioned in the report; and the Council gratefully record that it is largely owing to his experienced wisdom that the college has avoided certain familiar pitfalls, and secured the general esteem of the medical profession. A glance at the class of cases met with by the students, after their arrival in Africa, well shows the value of their training.

### Nature and Advertisement.

"This world is full of beauty, as angel worlds above," says the hymn, "and if we did our duty" in the matter of suppressing hideous advertisements, it might never lose its angelic characteristics. Such, at least, is the opinion of the Society for Checking the Abuses of Public Advertising. This society is engaged in the difficult task of trying to get rid of the ugly posters which not only cover the convenient hoardings in our towns, and smother the name of every railway station in large advices to buy soap and mustard, but obtrude themselves upon the humblest field by which the railway passes, and insult our lakes and mountains by their presence. We sympathise with the society, but we see little hope of their making any impression on the average advertiser. That energetic being may have a conscience of the ordinary moral kind, though on that point we do not pledge our editorial word; but an artistic conscience he certainly has not. Neither has he a sense of humour. If either gift were his he wouldn't do it. For it is ridiculous, as well as offensive, when one has climbed a mountain and is looking out on a glorious view, to be reminded, by a gigantic legend in the foreground, that Somebody's liver pills are worth ten pounds a box. At such a time one wants to forget that one has a liver. Neither is it fair when one is out on holiday that one should be tormented by such problems as "Why does a woman look old sooner than a man?" even though the explanation given be nothing more subtle than an assurance that lovely woman loses her looks because she does not use a certain soap. But so long as these advertisements induce people to buy the soap or the pills, so long will the announcements of their virtues meet us at every turn. But something might be done by appealing to the common-sense of the public. Know, gentle reader, that somebody must pay for these advertisements, and that somebody is you. The more numerous they are the more you pay, though, of course, there is the chance of more of you sharing the expense. But reflect, that advertising rarely pays unless the article advertised costs very little to produce, and perhaps you will look out for an equally good thing which you can buy at something nearer its worth. Not that any commodity can wholly do without advertisement. We cannot buy a thing if we have never heard of it; but there is a point at which legitimate advertising ceases, and further development become a shameless nuisance. Of these we have more than enough. At the same time, we must admit that there has recently been a great improvement in the artistic quality of advertising posters, although a few, in deserting the commonplace, have attained only a more incomprehensible ugliness. And the present writer is prepared to defy the convention that forbids painters willingly to paint posters. If a picture be good and well reproduced, it cannot be too widely circulated. It is bad work alone that dishonours the artist, whether it be displayed on a hoarding or on the walls of the Royal Academy.

